# Early hemoperfusion with an immobilized polymyxin B fiber column eliminates humoral mediators and improves pulmonary oxygenation

**DOI:** 10.1186/cc3815

**Published:** 2005-10-11

**Authors:** Hidehiko Kushi, Takahiro Miki, Kazuhiko Okamaoto, Jun Nakahara, Takeshi Saito, Katsuhisa Tanjoh

**Affiliations:** 1 Department of Emergency and Critical Care Medicine, Nihon University School of Medicine, 30-1 Oyaguchi Kamimachi, Itabashi-ku, Tokyo, 173-8610, Japan; 2 Department of Clinical Engineering, Surugadai Nihon University Hospital, 1-8-13 Kanda Surugadai, Chiyoda-ku, Tokyo, 101-8309, Japan

## Abstract

**Introduction:**

The objective of this study was to clarify the efficacy and mechanism of action of direct hemoperfusion with an immobilized polymyxin B fiber column (DHP-PMX) in patients with acute lung injury or acute respiratory distress syndrome caused by sepsis.

**Method:**

Thirty-six patients with sepsis were included. In each patient a thermodilution catheter was inserted, and the oxygen delivery index and oxygen consumption index were measured. DHP-PMX was performed in patients with a normal oxygen delivery index and oxygen consumption index (> 500 ml/minute per m^2 ^and >120 ml/minute per m^2^, respectively). The Acute Physiology and Chronic Health Evaluation II score was used as an index of the severity of sepsis, and survival was assessed after 1 month. The humoral mediators measured were the chemokine IL-8, plasminogen activator inhibitor-1, and neutrophil elastase (NE). These mediators were measured before DHP-PMX treatment, and at 24, 48, and 78 hours after the start of treatment. The arterial oxygen tension (PaO_2_)/fractional inspired oxygen (FiO_2_) ratio was measured before DHP-PMX treatment and at 24, 48, 72, 92, and 120 hours after the start of treatment.

**Results:**

All patients remained alive after 1 month. Before DHP-PMX treatment, the Acute Physiology and Chronic Health Evaluation II score was 24 ± 2.0, the IL-8 level was 54 ± 15.8 pg/ml, plasminogen activator inhibitor-1 was 133 ± 28.1 ng/ml, and NE was 418 ± 72.1 μg/l. These three humoral mediators began to decrease from 24 hours after DHP-PMX treatment, and the decline became significant from 48 hours onward. The PaO_2_/FiO_2 _ratio was 244 ± 26.3 before DHP-PMX treatment but improved significantly from 96 hours onward. There were significant negative correlations between the PaO_2_/FiO_2 _ratio and blood levels of NE and IL-8.

**Conclusion:**

The mechanism of action of DHP-PMX is still not fully understood, but we report the following findings. The mean blood levels of plasminogen activator inhibitor-1, NE, and IL-8 were significantly decreased from 48 hours after DHP-PMX treatment. The mean PaO_2_/FiO_2 _ratio was significantly improved from 96 hours after DHP-PMX treatment. Improvement in the PaO_2_/FiO_2 _ratio appeared to be related to the decreases in blood NE and IL-8 levels.

## Introduction

Since the American College of Chest Physicians and the Society of Critical Care Medicine proposed broad definitions of the terms 'sepsis' and the 'systemic inflammatory response syndrome' (SIRS) in 1992 [1], the concept of sepsis has changed dramatically. However, despite recent progress in new therapies, sepsis and septic shock are still major causes of multiple organ failure (MOF), and clinical outcomes remain unsatisfactory for patients with MOF [2]. Many clinicians have made great efforts to improve the prognosis of sepsis and SIRS. Despite this, clinical trials of anti-endotoxin monoclonal antibodies [3,4] and cytokine antagonists have proved unsuccessful in controlling either sepsis or SIRS [5].

In sepsis and SIRS a cascade of inflammatory responses leads to the production of proinflammatory cytokines, which are released from macrophages and monocytes after stimulation by lipopolysaccharide produced by Gram-negative bacteria or lipoteichoic acids produced by Gram-positive bacteria. These cytokines are importantly involved in the progression from sepsis to MOF. To inhibit this cascade of ongoing inflammatory reactions, a new therapy that eliminates the pathogenic toxins that trigger these reactions has been developed in Japan. A polymyxin B immobilized fiber column (PMX; Toray Industries Inc., Tokyo, Japan) was released in Japan in 1994 and it has been used for the treatment of endotoxemia. PMX therapy not only inhibits lipopolysaccharide production by Gram-negative bacteria but also lipoteichoic acid production by Gram-positive bacteria, as demonstrated in an experimental study [6]. It was reported in an experimental study [7] that removal of endotoxin with PMX can significantly improve survival rate, and the indications for PMX have gradually been defined.

However, Vincent and coworkers [8] found no difference in survival when they investigated the clinical benefit of direct hemoperfusion with PMX (DHP-PMX) in a randomized controlled study. On the other hand, in their randomized controlled study, Tani and colleagues [9] reported improved survival in patients with Acute Physiology and Chronic Health Evaluation (APACHE) II scores in the range 20–30. In an uncontrolled observational study [10] and a case report [11] DHP-PMX was used to treat peritonitis caused by colorectal perforation and acute cholecystitis, respectively, and PMX has proved to be of clinical benefit in these conditions. In their controlled observational study, Tsushima and coworkers [12] reported that PMX was able to improve significantly the arterial oxygen tension (PaO_2_)/fractional inspired oxygen (FiO_2_) ratio in patients with acute lung injury (ALI) or acute respiratory distress syndrome (ARDS) [13], although the mechanism of action was unclear. The incidence of ALI and ARDS is increased, and levels of blood humoral mediators are higher in patients with sepsis than in patients with trauma [14,15]. Thus, in addition to improving hemodynamics, improvement in pulmonary oxygenation in patients with ALI or ARDS has become an important goal during treatment for sepsis.

The following mechanisms have been suggested to explain the development of ALI and ARDS in patients with sepsis. Bacterial products such as endotoxin induce production of IL-8 by vascular endothelial cells and IL-8 activates neutrophils. As a result, the expression of adhesion molecules such as CD11/CD18 by activated neutrophils increases, as does the production of E-selectin and intercellular adhesion molecule-1 by pulmonary vascular endothelial cells. Neutrophils adhere to the pulmonary vascular endothelium by binding to these adhesion molecules, pass through the junctions between endothelial cells, and migrate into the extravascular space. Neutrophils cause damage to the pulmonary vascular endothelium by releasing oxygen radicals, proteinases, leukotrienes, and other proinflammatory molecules such as platelet-activating factor, thus impairing the barrier function of the pulmonary capillaries and leading to the onset of ALI or ARDS [16,17].

Based on the above mechanisms of development for ALI and ARDS, we formulated a hypothesis that the inhibitory effect of PMX on activation of chemokines, neutrophils, and vascular endothelial cells was associated with improvement in the PaO_2_/FiO_2 _ratio. In the present study we measured peripheral blood levels of IL-8, neutrophil elastase (NE), and plasminogen activator inhibitor (PAI)-1 (a marker of vascular endothelial cell activation) [18] over time to examine whether PMX could inhibit the production of these humoral mediators and improve the PaO_2_/FiO_2 _ratio.

## Materials and methods

### Selection of patients

This study was approved by the hospital ethics committee, and proper informed consent (oral or written) was obtained from each patient or their family. Patients with a clinical diagnosis of sepsis according to the criteria proposed by the American College of Chest Physicians/Society of Critical Care Medicine Consensus Conference were enrolled in the study [1]. Before the start of PMX treatment, global oxygen metabolism was measured. A thermodilution catheter (Edwards Life Sciences LLC, Irvine, CA, USA) was used to determine the oxygen delivery index and oxygen consumption index as parameters of oxygen metabolism. A thermodilution catheter was also used to monitor hemodynamics, and fluid balance was managed to maintain the central venous pressure in the range 7–10 mmHg. Criteria for inclusion in the study were the following findings within the previous 24 hours: signs of SIRS caused by infection (including fever or hypothermia [temperature > 38°C or < 36°C, respectively], tachycardia [> 90 beats/minute], tachypnea [> 20 breaths/minute], or an arterial carbon dioxide tension < 32 mmHg or mechanical ventilation, and a white blood cell count > 12.0 × 10^4^/l or < 4.0 × 10^4^/l or at least 10% immature neutrophils); mean arterial pressure > 60 mmHg irrespective of the use of catecholamines; stable global oxygen metabolism (oxygen delivery index > 500 ml/minute per m^2 ^and oxygen consumption index > 120 ml/minute per m^2^); and a diagnosis of ALI or ARDS according to the criteria established by the American-European Consensus Conference [13].

The diagnostic criteria for ALI and ARDS of the American-European Consensus Conference are as follows: acute onset of lung injury, diffuse bilateral infiltrates on chest X-ray film, a PaO_2_/FiO_2 _ratio < 200 mmHg for ARDS and < 300 mmHg for ALI, and a pulmonary artery occlusion pressure < 19 mmHg or no clinical evidence of congestive heart failure. Exclusion criteria were age < 18 years, mean arterial pressure ≤ 60 mmHg irrespective of the use of catecholamines, and impaired global oxygen metabolism.

The APACHE II score [19] was employed to assess of the severity of each patient's condition before DHP-PMX was performed. Survival of the patients was assessed at 1 month after treatment.

The three humoral mediators measured were IL-8 as a chemokine, PAI-1 as an index of vascular endothelial cell activation, and NE as an index of neutrophil activation. These mediators were measured before DHP-PMX treatment, and at 24, 48, and 72 hours after the start of therapy. Blood samples for the measurement of these mediators were collected from a peripheral vein. The neutrophil count was also determined. The PaO_2_/FiO_2 _ratio was used as an index of pulmonary oxygenation, and was measured before institution of DHP-PMX, and at 24, 48, 72, 92, and 120 hours after the start of treatment.

DHP-PMX was performed in 36 patients who had sepsis caused by Gram-positive or Gram-negative bacteria, and who fulfilled the inclusion criteria given above.

### Ventilation

Because they had ALI or ARDS, all of the patients included in the study needed mechanical ventilation; the tidal volume and plateau pressure were set at 6 ml/kg [20] and ≤ 30 cmH_2_O, respectively. The initial ventilation mode was volume-targeted assist control, which was changed to pressure-targeted assist control if the plateau pressure increased. The positive end-expiratory pressure (PEEP) was set at 5–15 cmH_2_O and the ventilation rate at 10–35 breaths/minute.

The ventilator protocol at our hospital involved FiO_2 _initially being set at 1.0 and PEEP at 10 cmH_2_O. Then PEEP was increased in 2 cmH_2_O increments up to 15 cmH_2_O if PaO_2 _remained below 55 mmHg on arterial blood gas analysis. When PaO_2 _was 80 mmHg or greater, FiO_2 _was decreased by 0.1. If PaO_2 _was 100 mmHg or greater, even after decreasing FiO_2 _to 0.6, then PEEP was decreased by 2 cmH_2_O. PEEP was discontinued at a PaO_2_/FiO_2 _ratio of 300 or greater, and PEEP under 5 cmH_2_O was not used.

Both ventilation and weaning from the ventilator were performed according to standardized protocols.

### Direct hemoperfusion

DHP-PMX was performed using a column of polystyrene fibers to which polymyxin B was covalently bound at a weight ratio of 0.5%. Binding of polymyxin B to the fibers was confirmed to be strong [7]. Blood access for DHP-PMX was obtained via a double-lumen catheter inserted into the femoral vein using Seldinger's method. Treatment was carried out for 3 hours at a flow rate of 80–100 ml/minute, and DHP-PMX was performed twice within 24 hours. Nafamostat mesilate (Torii Co., Ltd, Tokyo, Japan) was used as the anticoagulant. This is a serine protease inhibitor that exerts its anticoagulant effect primarily by inhibiting thrombin. The half-life of nafamostat mesilate is 8 minutes, and so its anticoagulant effect was confined to the extracorporeal circuit.

### Plasminogen activator inhibitor-1 assay

PAI-1 was measured in duplicate by an enzyme-linked immunosorbent assay (Fuji Revio Inc., Tokyo, Japan). PAI-1 is known to have various forms in blood, including an active form, an inactive form, a latent form, tissue plasminogen activator/PAI-1 complex, and vitronectin/PAI-1 complex. We measured total PAI-1 in the present study to determine the total amount of PAI-1 produced by vascular endothelial cells.

### Measurement of neutrophil elastase

The blood level of NE was measured by using a commercial enzyme immunoassay kit (Sanwa Chemical Institute, Nagoya, Japan). Two forms of NE (free NE and NE/α_1_-antitrypsin complex) can be detected using the kit. The normal NE level, determined in 139 healthy persons using this kit, was found to be 29 ± 2.7 μg/l (SRL Inc., Tokyo Japan, unpublished data).

### Measurement of IL-8

Four blood samples were collected from a peripheral vein. After centrifugation, serum was stored for a maximum of one month at -70°C. Then the serum level of IL-8 was determined using a commercially available specific enzyme-linked immunosorbent assay kit for IL-8 (Ohtsuka Pharma Co., Tokyo, Japan), in accordance with the manufacturer's instructions.

### Statistical analysis

Results are expressed as mean ± standard error. The unpaired Wilcoxon's test or one-way analysis of variance was used to determine statistically significant differences. *P *< 0.05 was considered statistically significant. The correlation coefficient was obtained using Spearman's equation.

## Results

DHP-PMX was performed 72 times in 36 patients (21 men and 15 women), aged 28–85 years old (mean 62 ± 18.5 years). Treatment was performed twice (for 3 hours/session) within a 24-hour period. As shown in Table 1, the patients had various underlying diseases and had multiple conditions. Table 2 shows the sources of infection; pneumonia was the most frequent type of primary infection. Table 3 shows the causative organisms. The most commonly isolated microorganisms were Gram-negative bacteria, and 48 bacterial strains were detected in the 36 patients. Antibiotic therapy was judged to be adequate when the patient received drugs to which each isolated microorganism was sensitive. Although it was not possible to identify bacteria in two patients, cultures were positive in 34 patients and adequate antibiotic treatment was given to them.

The APACHE II score was 24 ± 2.0 before DHP-PMX. (Propofol was used as a sedative, and it was temporarily discontinued from 06:00 to 08:00 hours and the Glasgow Coma Scale score was evaluated thereafter.) The mean PaO_2_/FiO_2 _ratio was 244 ± 26.3 and the mean level of PEEP was 8 ± 1.3 cmH_2_O at the start of DHP-PMX treatment. Because PEEP was discontinued at a PaO_2_/FiO_2 _ratio of 300 or above, its mean duration was approximately 81 ± 13.5 hours.

**Figure 1:**
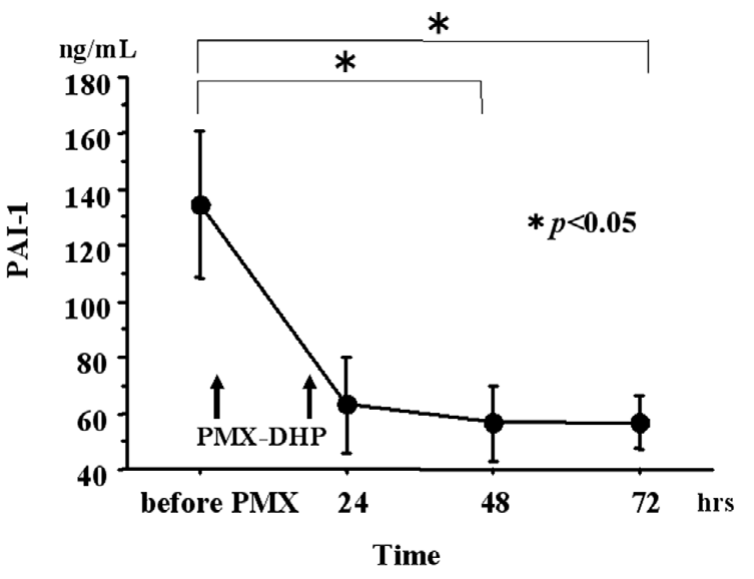
Changes in PAI-1. The plasminogen activator inhibitor (PAI)-1 level was 1,138 ± 26.3 ng/ml before direct hemoperfusion with an immobilized polymyxin B fiber column (DHP-PMX), but it was 67 ± 17.4 ng/ml at 24 hours after the start of treatment, 54 ± 13.6 ng/ml at 48 hours, and 52 ± 9.8 ng/ml at 72 hours, with statistically significant decreases from 48 hours onward compared with baseline (*P *< 0.05).

The PAI-1 level was 133 ± 28.1 ng/ml before DHP-PMX treatment, whereas it was 67 ± 17.4 ng/ml at 24 hours after the start of treatment, 54 ± 13.6 ng/ml at 48 hours, and 52 ± 9.8 ng/ml at 72 hours, with decreases being statistically significant from 48 hours onward compared with baseline (*P *< 0.05; Fig. 1).

**Figure 2:**
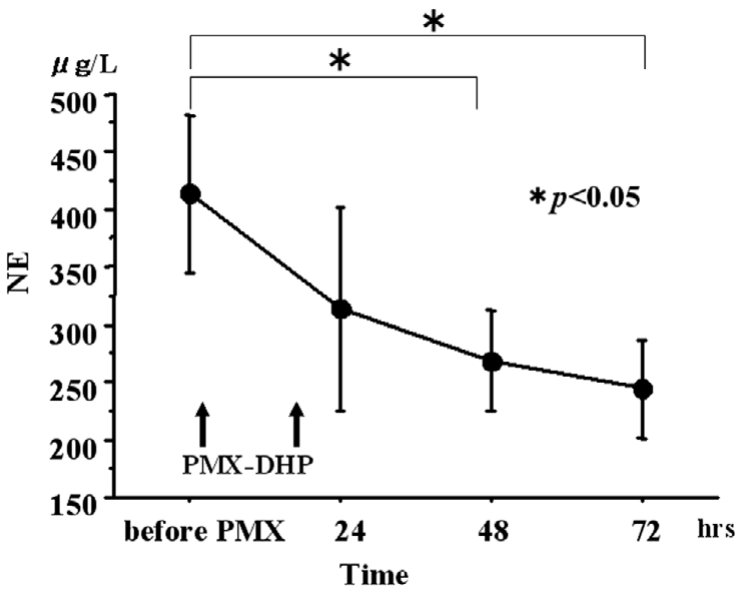
Changes in NE. The neutrophil elastase (NE) level was 443 ± 68.2 μg/l before direct hemoperfusion with an immobilized polymyxin B fiber column (DHP-PMX), but it was 366 ± 74.0 μg/l at 24 hours after the start of treatment, 274 ± 42.5 μg/l at 48 hours, and 249 ± 41.3 μg/l at 72 hours, with statistically significant decreases from 48 hours onward compared with baseline (*P *< 0.05).

The NE level was 418 ± 72.1 μg/l before DHP-PMX treatment, whereas it was 366 ± 74.0 μg/l at 24 hours after the start of treatment, 274 ± 42.5 μg/l at 48 hours, and 249 ± 41.3 μg/l at 72 hours, with decreases being statistically significant from 48 hours onward compared with baseline (*P *< 0.05; Fig. 2). The mean neutrophil count was 14.157 ± 2.1785 × 10^9^/l before DHP-PMX treatment, but it was 13.054 ± 1.816 × 10^9^/l at 24 hours after the start of treatment, 13.343 ± 1.3978 × 10^9^/l at 48 hours, and 12.206 ± 1.753 × 10^9^/l at 72 hours; differences from baseline were not statistically significant.

**Figure 3:**
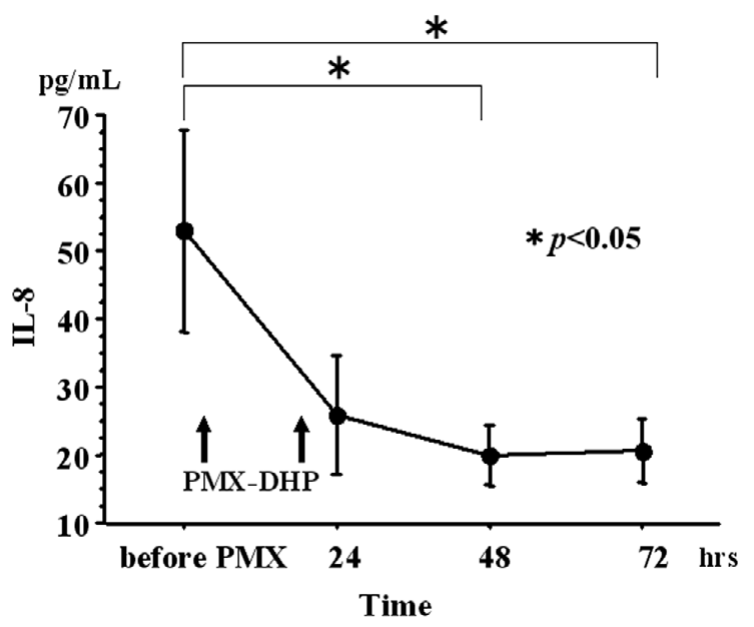
Changes in IL-8. The IL-8 level was 54 ± 14.6 pg/ml before direct hemoperfusion with an immobilized polymyxin B fiber column (DHP-PMX), but it was 27 ± 7.5 pg/ml at 24 hours after the start of treatment, 19 ± 4.3 pg/ml at 48 hours after, and 20 ± 4.5 pg/ml at 72 hours after, with a statistically significant decrease from 48 hours onward compared with baseline (*P *< 0.05).

The IL-8 level was 54 ± 15.8 pg/ml before DHP-PMX treatment, but it was 27 ± 7.5 pg/ml at 24 hours after the start of treatment, 19 ± 4.3 pg/ml at 48 hours, and 20 ± 4.5 pg/ml at 72 hours, with statistically significant decreases compared with baseline from 48 hours onward (*P *< 0.05; Fig. 3).

**Figure 4:**
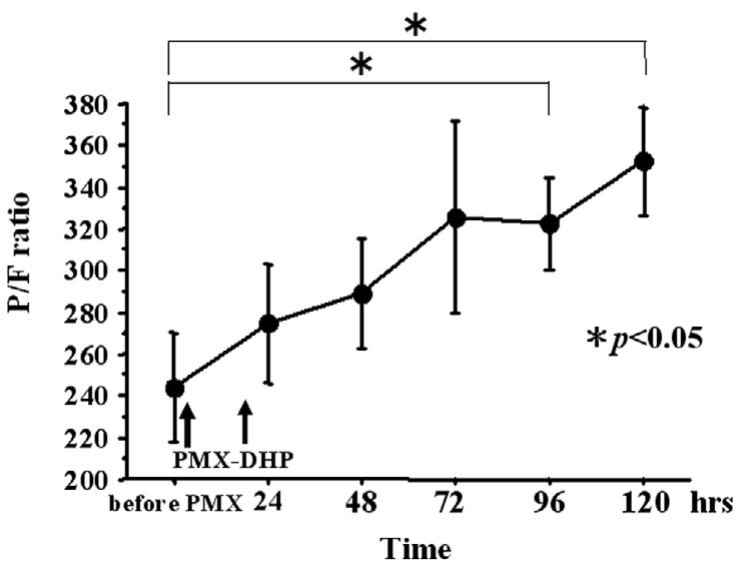
Changes in PaO_2_/FiO_2 _ratio. The arterial oxygen tension (PaO_2_)/fractional inspired oxygen (FiO_2_) ratio was 244 ± 26.3 before direct hemoperfusion with an immobilized polymyxin B fiber column (DHP-PMX). It increased to 274 ± 28.6 at 24 hours after the start of treatment, 289 ± 26.2 at 48 hours, 348 ± 46.0 at 72 hours, 322 ± 22.1 at 96 hours, and 352 ± 25.4 at 120 hours, with statistically significant improvement from 96 hours onward compared with baseline (*P *< 0.05).

The PaO_2_/FiO_2 _ratio was 244 ± 26.3 before DHP-PMX treatment. It increased to 274 ± 28.6 at 24 hours afterward the start of treatment, 289 ± 26.2 at 48 hours, 348 ± 46.0 at 72 hours, 322 ± 22.1 at 96 hours, and 352 ± 25.4 at 120 hours, with significant improvements compared with baseline from 96 hours onward (*P *< 0.05; Fig. 4).

**Figure 5:**
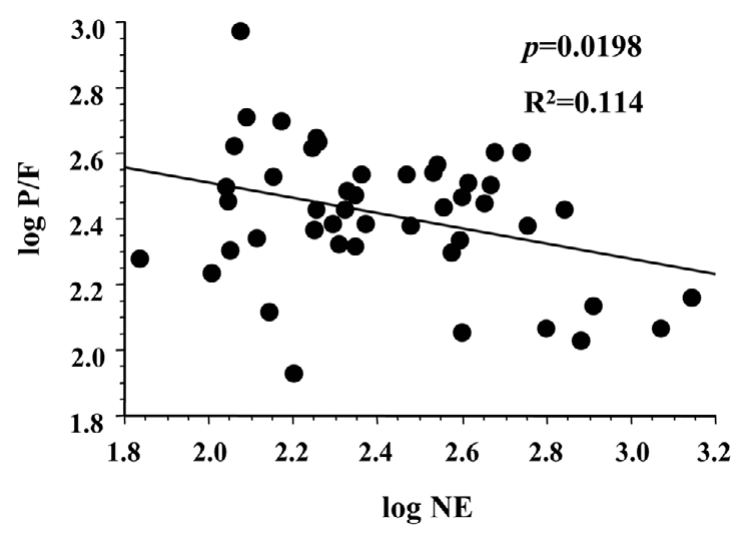
Relationship between the PaO_2_/FiO_2 _ratio and NE. There was an inverse relationship between the arterial oxygen tension (PaO_2_)/fractional inspired oxygen (FiO_2_) ratio (P/F ratio) and peripheral blood neutrophil elastase (NE) level. The correlation coefficient between the log-transformed PaO_2_/FiO_2 _ratio and log-transformed NE value was -0.337 (*P *= 0.0198).

**Figure 6:**
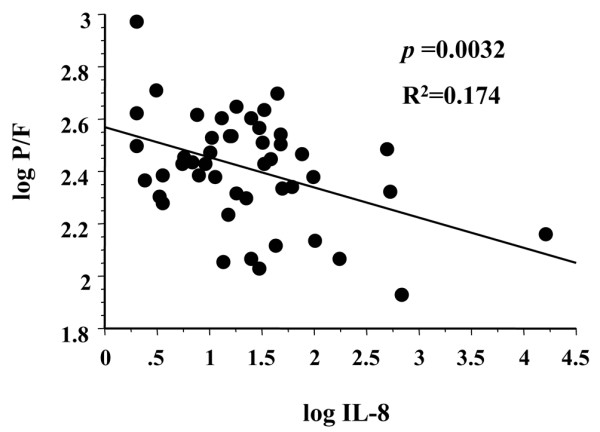
Relationship between the PaO_2_/FiO_2 _ratio and IL-8. There was an inverse relationship between the arterial oxygen tension (PaO_2_)/fractional inspired oxygen (FiO_2_) ratio (P/F ratio) and peripheral blood IL-8 level. The correlation coefficient between the log-transformed PaO_2_/FiO_2 _ratio and log-transformed IL-8 value was -0.417 (*P *= 0.0032).

The correlation coefficients (r values) for the relationships between the PaO_2_/FiO_2 _ratio and each of the humoral mediators were as follows: -0.186 (*P *= 0.216) between the log-transformed PaO_2_/FiO_2 _ratio and log-transformed PAI-1 value; -0.337 (*P *= 0.0198) between the log-transformed PaO_2_/FiO_2 _ratio and log-transformed NE value; and -0.417 (*P *= 0.0032) between the log-transformed PaO_2_/FiO_2 _ratio and log-transformed IL-8 value. Thus, there were statistically significant inverse relationships between the PaO_2_/FiO_2 _ratio and peripheral blood levels of NE and IL-8 (Figs 5 and 6).

## Discussion

### Patient selection

No standard criteria for the use of DHP-PMX have been established. It has been variously reported that an IL-6 level of 1,000 pg/ml or greater [21], an APACHE II score of 25 or greater [22], an APACHE II score of 30 or greater [9], and a cardiac index of 6 l/minute per m^2 ^or greater [23] are indicators of poor prognosis at the start of DHP-PMX treatment. The reported efficacy of DHP-PMX also varies among medical institutions, depending on the time of starting treatment. In the present study, DHP-PMX was performed before the deterioration of organ perfusion and global oxygen metabolism. Our use of criteria targeting global oxygen metabolism and the mean blood pressure was based on the concept that DHP-PMX should be started before the tissues and cells have been significantly affected, even if some organ disfunction is present. Once shock occurs and organ ischemia progresses, cellular disfunction at the molecular level becomes severe, and this makes it difficult to assess the effect of DHP-PMX. Therefore, we selected patients for this therapy according to criteria that excluded those already in shock. It is perhaps a result of the early introduction of DHP-PMX before oxygen metabolism had deteriorated that all of the patients remained alive after 1 month.

### Plasminogen activator inhibitor-1

PAI-1 is a protein with a molecular weight of 50 kDa that is produced by vascular endothelial cells and plays a central role in the activation of fibrinolysis [24]. It has recently attracted attention as a marker of vascular endothelial cell activation [18]. We measured total PAI-1 in the present study to determine the total production of this mediator by vascular endothelial cells.

Proinflammatory cytokines such as TNF-α and IL-1β stimulate vascular endothelial cells, induce the expression of adhesion molecules, and cause activated neutrophils to stimulate PAI-1 production by vascular endothelial cells, thus inhibiting fibrinolysis [18]. In the present study, PAI-1 was significantly lower at 48 and 72 hours after the start of DHP-PMX then the baseline value. Rather than DHP-PMX directly inhibitiing PAI-1 production, it seems more likely that adsorption of pathogenic bacteria prevented the release of inflammatory cytokines and reduced the stimulation of vascular endothelial cells to lower the PAI-1 level indirectly.

### Neutrophil elastase

NE is a glycoprotein with a molecular weight of approximately 30 kDa and it has three isozymes. Its physiologic actions are proteolysis of bacteria and foreign materials. Because of low substrate specificity, however, NE can also attack various host proteins such as plasma proteins, coagulation factors, complement, elastin, and collagen [25]. Intact cells can also be damaged by this protease [26]. Neutrophils are believed to be an important precipitating factor in the pathogenesis of ARDS [16,17]. Indeed, neutrophils may contribute to the development of lung disfunction by releasing oxygen radicals and proteolytic enzymes that induce the parenchymal cell damage and connective tissue destruction characteristic of ARDS [16,17].

We investigated changes in circulating NE following DHP-PMX and found that blood levels after 48 and 72 hours were significantly lower than before treatment. Adsorption of pathogenic toxins may have prevented the release of inflammatory cytokines [22], resulting in less stimulation of neutrophils, rather than DHP-PMX directly preventing cytokine production or neutrophils being cleared from the blood by the column, because the neutrophil count was not altered by this treatment. Thus, DHP-PMX appears to prevent the activation of neutrophils, albeit indirectly. The ability of DHP-PMX to prevent activation of vascular endothelial cells [27] and reduce the blood level of NE might help to decrease organ disfunction, and the same mechanisms are considered to be associated with improvement in PaO_2_/FiO_2 _ratio in the patients with ALI and ARDS studied here.

### Interleukin-8

IL-8 is a 6.5 kDa cytokine [28] that belongs to a supergene family of host defense molecules characterized by potent neutrophil activation *in vitro *[29]. It can be produced by several types of cells *in vitro*, including macrophages, lymphocytes, neutrophils, fibroblasts, and endothelial cells, in response to a variety of stimuli such as endotoxin, viruses, and other cytokines such as IL-1 or tumor necrosis factor [29]. Similar to PAI-1 and NE, the mean blood level of IL-8 was significantly decreased from 48 hours after DHP-PMX compared with the baseline value. This observation suggests that removal of pathogenic toxins from the blood indirectly inhibited the release of IL-8 from IL-8 producing cells, as was observed for PAI-1 and NE. In fact, an *in vitro *study [30] has demonstrated that absorption of inflammatory cytokines by PMX is quite limited. However, there has been no previous report on the effect of this treatment on IL-8. The results of the present study suggest that the inhibitory effect of DHP-PMX on IL-8, which is a key mediator of inflammatory reactions [31], may contribute to improvement in PaO_2_/FiO_2 _ratio.

Ventilation parameters may be another factor that influences circulating IL-8 concentration. Parsons and coworkers [32] reported that mechanical ventilation with a lower tidal volume of 6 ml/kg reduced the mean blood IL-8 level by approximately 12% in patients with ALI on the third day after the onset, suggesting that the decrease of blood IL-8 levels observed in our study was possibly associated with mechanical ventilation at low tidal volumes. However, the mean blood IL-8 level was decreased by approximately 72% compared with baseline on day 3 after the onset of treatment, which may represent a synergistic effect between mechanical ventilation at low tidal volumes and DHP-PMX.

### Relationship between the PaO_2_/FiO_2 _ratio and humoral mediators

During ventilation, PEEP and fluid balance are factors that are likely to influence pulmonary oxygenation, other than humoral mediators. The mean PaO_2_/FiO_2 _ratio was 244 ± 26.3 and the mean level of PEEP was 8 ± 1.3 cmH_2_O at the start of DHP-PMX. Because PEEP was discontinued at a PaO_2_/FiO_2 _ratio of 300 or greater, its mean duration was approximately 81 ± 13.5 hours. The level of PEEP was gradually reduced during this period, and PEEP of 5 cmH_2_O or less was not employed. Given that the PaO_2_/FiO_2 _ratio improved while PEEP was gradually reduced, it is not likely that PEEP directly contributed to the improvement in pulmonary oxygenation. A thermodilution catheter was inserted before the start of treatment to monitor oxygen metabolism and hemodynamics for 7 days. Because the central venous pressure was maintained between 7 and 10 mmHg in all patients, it is not likely that the fluid balance had a direct influence on differences in pulmonary oxygenation.

Tsushima and coworkers [12] reported that the PaO_2_/FiO_2 _ratio was significantly improved at 2 hours after DHP-PMX in all 20 patients with ALI or ARDS that they treated. They suggested that the observed improvement in PaO_2_/FiO_2 _ratio was related to inhibition of the production of inflammatory mediators and consequent suppression of systemic inflammation, both of which are caused by modulation of activated mononuclear cells and neutrophils by PMX, although the detailed mechanism of action remains unknown.

In the present study, although the PaO_2_/FiO_2 _ratio was significantly improved from 96 hours after DHP-PMX, no improvement was observed soon after treatment (24 hours). The patients included had definite ALI and/or ARDS and did not have cardiogenic pulmonary edema, according to assessment with a thermodilution catheter before DHP-PMX. The following mechanisms underlying the development of ALI and ARDS in patients with sepsis have been suggested. Bacterial substances such as endotoxin induce the production of IL-8 by stimulating pulmonary vascular endothelial cells and thus activate neutrophils. Production of adhesion molecules is then accelerated, causing neutrophils to adhere to pulmonary vascular endothelial cells and migrate into the extravascular space. Neutrophils accumulate locally in the lungs along the concentration gradients of chemotactic factors such as IL-8. Neutrophils then cause damage to pulmonary vascular endothelial cells and impair the barrier function of pulmonary capillaries, leading to the development of ALI or ARDS [16,17]. Considering the above mechanisms, it is unlikely that PaO_2_/FiO_2 _ratio would improve immediately after DHP-PMX.

In the present study we examined correlations between the PaO_2_/FiO_2 _ratio and circulating levels of mediators that are considered to be associated with the development of ARDS (i.e. PAI-1, NE, and IL-8). Our findings indicate that there are significant negative correlations between the PaO_2_/FiO_2 _ratio and IL-8 and NE levels. Jorens and coworkers [33] reported a negative correlation between the PaO_2_/FiO_2 _ratio and the IL-8 level in bronchoalveolar lavage fluid as well as between the PaO_2_/FiO_2 _ratio and infiltration of neutrophils in patients with ARDS. Although the correlations demonstrated in the present study did not indicate such strong relationships, perhaps because we measured peripheral blood levels, the results of our study are consistent with the findings of Jorens and coworkers.

We used PAI-1 as an index of vascular endothelial cell activation, but no significant relationship with the PaO_2_/FiO_2 _ratio was identified, probably resulting from testing of peripheral blood samples. If we had measured the PAI-1 level in bronchoalveolar lavage fluid, then a significant correlation with the PaO_2_/FiO_2 _ratio might have been demonstrated. The reasons why a period of 96 hours is required to detect improvement in the PaO_2_/FiO_2 _ratio is that the patients had definite ARDS or ALI, and that 48 hours after DHP-PMX were required for decreases in PAI-1, NE, and IL-8 concentrations to occur.

Because this work is an uncontrolled observational study, observed changes can not be concluded to result from extracorporeal treatment. It will be necessary to conduct controlled studies in this area in the future.

## Conclusion

The mechanism of action of DHP-PMX is not fully understood, but DHP-PMX significantly decreased blood levels of PAI-1, NE and IL-8, and significantly improved the PaO_2_/FiO_2 _ratio. There were significant negative correlations between the PaO_2_/FiO_2 _ratio and IL-8 and NE levels. The present study showed that early performance of DHP-PMX eliminated humoral mediators and improved pulmonary oxygenation.

This was a prospective uncontrolled observational study including a limited number of patients. The results of larger, better powered, multicenter clinical trials are necessary if we are to assess accurately the benefit of DHP-PMX.

## Key messages

• 	The mean blood levels of PAI-1, NE, and IL-8 were significantly decreased from 48 hours after start of DHP-PMX treatment.

• 	The mean PaO_2_/FiO_2 _ratio was significantly improved from 96 hours after the start of DHP-PMX treatment.

• 	There were significant negative correlations between the PaO_2_/FiO_2 _ratio and blood levels of NE and IL-8.

• 	Improvement in PaO_2_/FiO_2 _ratio appeared to be related to the decreases in blood NE and IL-8 levels.

## Abbreviations

ALI = acute lung injury; APACHE = Acute Physiology and Chronic Health Evaluation; ARDS = acute respiratory distress syndrome; DHP-PMX = direct hemoperfusion with an immobilized polymyxin B fiber column; FiO_2 _= fractional inspired oxygen; IL = interleukin; MOF = multiple organ failure; NE = neutrophil elastase; PAI = plasminogen activator inhibitor; PaO_2 _= arterial oxygen tension; PEEP = positive end-expiratory pressure; SIRS = systemic inflammatory response syndrome.

## Competing interests

The authors declare that they have no competing interests.

## Authors' contributions

HK designed the study, processed the data, and wrote the manuscript. TM, KO, JN, and TS collected the clinical data. KT participated in designing the study. All authors read and approved the final manuscript.

## Figures and Tables

**Table 1:** Demographic characteristics and underlying diseases of the 36 patients

Characteristic	Value
Patients (*n*)	36
Age (years)	62 ± 18.5 (range 28–85)
Sex (male/female; *n*)	21/15
Underlying disease (*n*)	
Respiratory disease	11
Neurological disease	9
Cardiovascular disease	6
Diabetes mellitus	4
Recent trauma	5
Chronic liver disease	1

**Table 2:** Locations of infection

Location	*n*
Respiratory system	12
Abdominal cavity	6
Central nervous system	6
Urinary tract	4
Biliary tract	3
Cardiovascular system	3
Others	2

**Table 3:** Causative organisms

Organisms	*n*
Gram-negative bacteria	
*Pseudomonas aeruginosa*	11
*Escherichia coli*	8
*Klebsiella pneumoniae*	6
*Neisseria *spp.	3
*Vibrio *and *Citrobactor *spp.	1
Gram-positive bacteria	
*Staphylococcus aureus*	7
α-Streptococcus	4
*Staphylococcus epidermidis*	3
*Corynebacterium *spp.	1
*Enterococcus faecalis*	1
Fungi	
*Candida albicans*	3
